# Association between RNA-binding protein *Ptbp2* and germ cell injury in an experimentally-induced unilateral cryptorchidism murine model

**DOI:** 10.1371/journal.pone.0186654

**Published:** 2017-10-18

**Authors:** Xianming Dou, Jingjing Gao, Pan Gao, Dongdong Tang, Dangwei Peng, Jun Mao, Zhenyu Huang, Peng Chen, He Chen, Shengwei Ke, Chaozhao Liang, Xiansheng Zhang

**Affiliations:** 1 Department of Urology, the First Affiliated Hospital of Anhui Medical University, Hefei, Anhui, China; 2 Department of Urology, the Second Affiliated Hospital of Wannan Medical College, Wuhu, Anhui, China; 3 Department of Laboratory, the First Affiliated Hospital of Anhui Medical University, Hefei, Anhui, China; 4 Department of Cell and Developmental Biology, School of Life Sciences, University of Science and Technology of China, Hefei, Anhui, China; Centre de Recherche en Cancerologie de Lyon, FRANCE

## Abstract

RNA binding protein polypyrimidine tract binding protein 2 (Ptbp2) as a key alternative splicing regulator for male germ cell development is well established. However, its expression levels and role in cryptorchidism testes tissues has not been explored. Additionally, the molecular mechanism of heat stress impacts the correct proliferation and differentiation of germ cells is unclear. To investigate whether changes in Ptbp2 expression are correlated with heat stress-induced germ cell injury in testicular tissue, we used a murine model of intraperitoneal cryptorchidism with surgical operation. Here we present compelling evidence that germ cells are severely damaged in mice with unilateral cryptorchidism, with non-obstructive azoospermia. And the Ptbp2 and Pgk2 mRNA levels were significantly decreased in parallel, leading us to conclude that the negative correlation between Ptbp2 levels and germ cell injury in unilateral cryptorchidism murine model. We hypothesize that Ptbp2 is susceptible to heat stress and its disruption has resulted in stability decline of germ cell transcripts Pgk2 mRNA, which consequently lead to germ cell injury in cryptorchidism testes. Thus, we confirm that Ptbp2 is an essential factor in heat stress-induced sperm cell injury and non-obstructive azoospermia.

## Introduction

The proportion of infertility occurring among couples at childbearing age is about 10–15%, and 50% of these cases are thought to be due to male infertility [1]. Idiopathic diseases of the male reproductive system are important factors that can lead to male infertility. Cryptorchidism, or undescended testes, is characterized by the failure of one or both testes to descend to a normal scrotal position, and is the most common cause of non-obstructive azoospermia in adult males. The incidence of cryptorchidism is thought to be around 2–4% worldwide, and the testes of approximately 80% of cryptorchidism patients descend to a normal scrotal position within the first year after birth [2]. The current guidelines recommend orchiopexy intervention before 12 months of age to maximize fertility [3]. Nevertheless, from the beginning to 12 months after birth, germ cell injury in the cryptorchidism testes has initiated [4–6]. If left untreated, or if orchiopexy is performed too late, the incidence of male infertility due to cryptorchidism increases considerably [5,7]. It is generally accepted that heat stress induced by the high temperature of the abdominal cavity affects the proliferation and differentiation of germ cells in these individuals [8]. We suspect that some crucial signaling pathways of spermatogenesis may be particularly susceptible to heat stress, and their disruption may linked to dyszoospermia in the intraperitoneal cryptorchidism testes. This aberrant testicular environment has been shown to have a detrimental effect on testicular germ cell development, which may lead to non-obstructive azoospermia and testicular atrophy [3].

In recent decades, RNA-binding proteins (RBPs) have been identified as essential factors in post-transcriptional regulatory events, and have been increasingly recognized as necessary for spermatogenesis and testicular germ cell functions [9–12]. The polypyrimidine tract-binding (PTB) protein is a member of the hnRNP family of RBPs. Polypyrimidine tract-binding protein 2 (Ptbp2), a subtype of the PTB proteins, is an important transcription factor that regulates alternative splicing during male germ cell development, and also plays a vital function in cell proliferation and apoptosis [13,14]. In cases of Ptbp2 inactivation in the testes, the differentiation of germ cells arrests in the stage of round spermatids, resulting in the proliferation of multinucleated cells in the seminiferous tubule, increased apoptosis of spermatocytes, atrophy of seminiferous tubules, and a lack of elongating spermatids, which consequently affects male fertility.

Previous research has shown that Ptbp2 regulates neural precursor cell differentiation, and is abundantly expressed in the brain [15]. Ptbp2 is a multifunctional RBP with four RNA recognition motifs (RRM1 to RRM4), which bind to target mRNA sequences containing one or more pyrimidine-rich elements involved in mRNA stability, localization, alternative splicing and initiation of IRES(internal ribosomal entry site)-directed translation [16]. In germ cells, Ptbp2 plays an important role in the post-transcriptional control of target mRNAs, such as Pgk2 mRNA. The mRNA encoding the testes-specific phosphoglycerate kinase 2 protein is a long-lived mRNA which is essential for sperm function and male fertility [17,18]. In addition, *in vitro* transfection assays have demonstrated that Ptbp2 stabilizes the Pgk2 mRNA by directly binding to the F1 region of the 3'-UTR [16,19,20].

Despite the extensive studies focused on the function of Ptbp2 during spermatogenesis, no reports have been published on the behavior of Ptbp2 in idiopathic diseases of the reproductive system, especially cryptorchidism. To elucidate whether the changes in Ptbp2 expression are correlated with sperm cell injury and non-obstructive azoospermia in heat stress-affected testicular tissue, we investigated testicular tissues in a surgically-induced intraperitoneal cryptorchidism model in mice.

## Materials and methods

### Animals

Mice were obtained from the Laboratory Animal Center of Anhui Medical University and kept in a specific pathogen-free (SPF) animal facility. The mice were exposed to a 12-hour light/dark cycle and an ambient temperature of 22 ± 2°C, with free access to laboratory chow and water.

Male ICR mice, all 6-weeks-old, were randomly divided into two groups. The unilateral cryptorchidism (UC) group (n = 25) were anesthetized with 10% chloralic hydras (3 ml/kg administered intraperitoneally), then the skin of the left abdomen was prepared and disinfected. The artificial model of unilateral cryptorchidism was created by opening the abdominal cavity, pushing the left testes slightly into the peritoneal cavity, then clipping the gubernaculum and fixing the fat pad onto the peritoneum in order to simulate cryptorchidism (CRY) testes. The right testes was kept in the original position as the self-control (SC) testes. The sham-operated (SO) group (n = 25) were subjected to the method described above, but the left testes was kept in the original position. When the groups had reached 3, 7, 14, 21 and 28 days postoperation (dpo), mice were sacrificed by cervical dislocation and the testes and epididymides tissues were obtained and weighed. The epididymides were longitudinally cut and placed in 1 ml of phosphate-buffered saline (PBS) that had been prewarmed to 37°C. The sperm were allowed to swim out for 30 min prior to analysis of semen parameters. The testicular samples were divided into three parts, one third was fixed and embedded for histological examination, another third was placed in RNAfixer for quantitative real-time PCR, and the remaining testicular tissues were cryopreserved at -80°C for protein analysis. All mice experiments were performed in accordance with the relevant guidelines and regulations. This study received ethical approval from the institutional review board of Anhui Medical University(No.LLSC20150346).

### Western blot analysis

Western blot experiments were performed to detect the expression levels of the Ptbp2 protein in the testicular tissues of unilateral cryptorchidism mice. The proteins were lysed from the testicular samples following the method described by Wang et al. [21]. The protein lysates were loaded into SDS page gels, then electroblotted onto nitrocellulose membranes. Membranes were blocked for non-specific binding by immersing the membrane in 5% fat-free milk in TBST (10 mM Tris [pH 7.5], 200 mM NaCl and 0.2% Tween 20) for 1 hour at room temperature with shaking. This was followed by incubation with primary antibodies (anti-PTBP2, diluted 1:1000) and the secondary antibody (anti-GAPDH, diluted 1:2000). The anti-PTBP2 and anti-GAPDH antibodies used for western blot analysis were purchased from Abcam (Cambridge, MA, USA).

### Histological and immunohistochemical analyses

To assess the degree of damage to the sperm cells, hematoxylin and eosin (HE) staining was carried out to evaluate testicular histological changes in the mouse model. In addition, localization of the Ptbp2 protein in the testicular tissues of mice was determined by immunohistochemistry (IHC). The testicular samples were fixed with 4% paraformaldehyde, then embedded in paraffin wax and sectioned at 4 μm. The testicular tissue sections were deparaffinized in different concentrations of dimethylbenzene and ethanol solutions, then heated in sodium citrate buffer (pH 6.0) in a microwave for 20 min. Next, the sections were dipped in deionized water containing 3% H_2_O_2_ to quench endogenous peroxidase activity. After specific treatment with 10% normal goat serum to block non-specific binding, the deparaffinized sections were incubated overnight at 4°C with the anti-PTBP2 antibody (Abcam) at 1:300 dilution, then incubated with a goat anti-rabbit biotinylated secondary antibody for 40 min at room temperature. The immunoreactivity with Ptbp2 was observed using streptavidin-peroxidase and 3,3N-diaminobenzidine (Beyotime, Jiangsu, China).

### Quantitative real-time PCR

Total RNA was extracted from the testicular samples and subjected to quantitative real-time PCR. RNA was isolated using the trizol protocol according to the standard procedure.

The primers were as follows: Pgk2, forward 5′- CCATCCCAAGTATCAAGC -3′ and reverse 5′- TCCAGCAGGATGATAGACCC -3′; and β-actin, forward 5′- CGTTGACATCCGTAAAGACC -3′ and reverse 5′- AACAGTCCGCCTAGAAGCAC -3′.

The quantitative real-time PCR was carried out as previously described [22]. We used the melting-curve analysis to monitor the purity of the PCR product, and the relative expression of Pgk2 mRNA was normalized to β-actin.

### Sperm analysis

To analyze the sperm parameters, the entire epididymides[23] from the mice were longitudinally cut and placed in 1 ml PBS that had been prewarmed to 37°C, and sperm were allowed to swim out for 30 min. The sperm density and motility outcomes were manually evaluated using a Computer-Assisted Semen Analysis (CASA) system (WLJY-9000, China).

### Statistical analysis

All data analyses were performed with the SPSS software, version 13.0 (SPSS Inc, Chicago, IL, USA). Data are presented as mean ± standard deviation(s.d.). A p-value < 0.05 was considered statistically significant in all analyses.

## Results

**Fig 1:**
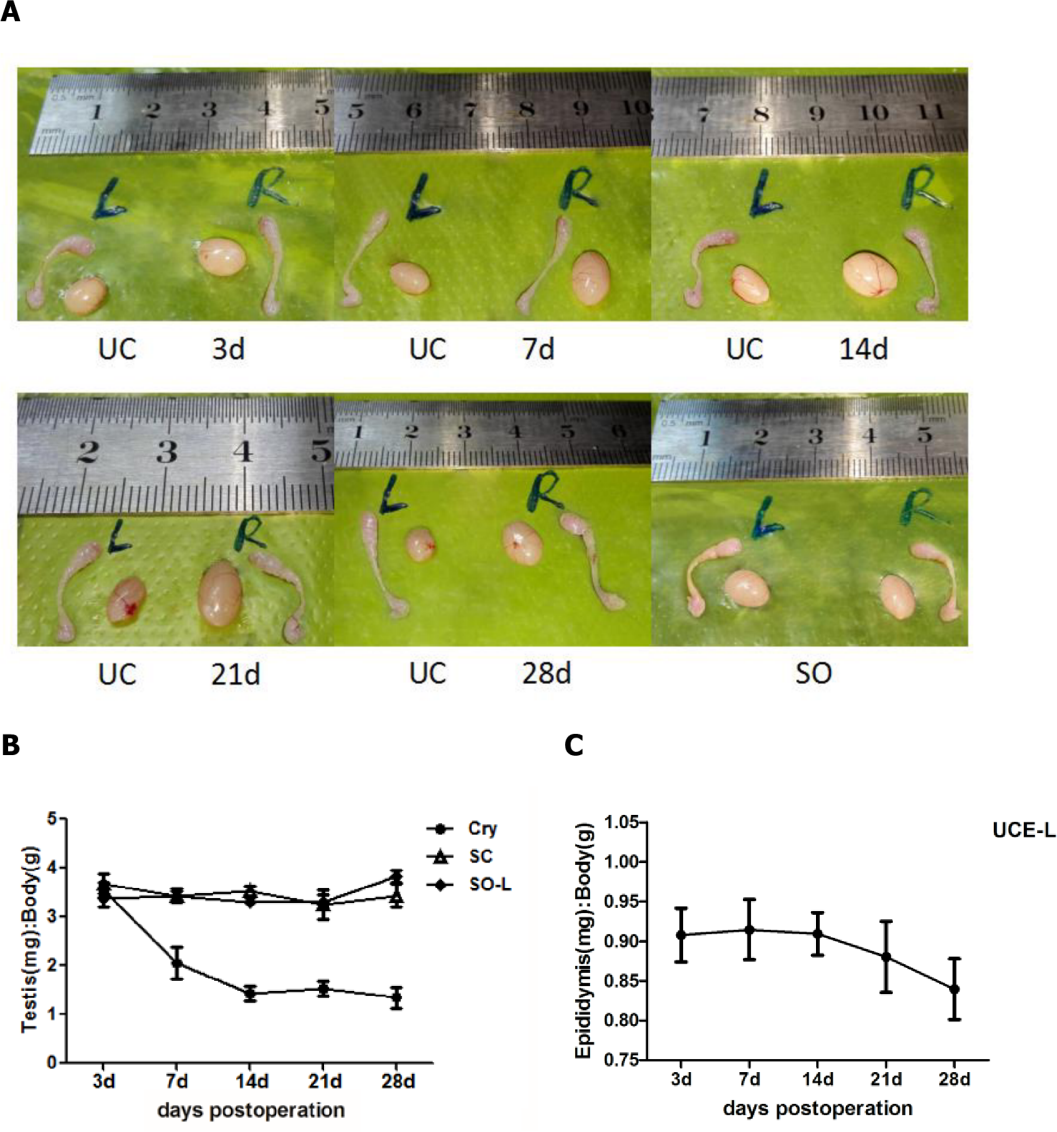
(A) Photographs of testes and epididymis of mice with UC and SO groups. (B) The testes index (ratio = testes weight/body weight) of the testes of CRY was downregulated compared with that of SC and SO-L at 7, 14, 21 and 28 days postoperation. (C) The epididymides index (ratio = epididymides weight/body weight) changes in the left epididymides of UC group. Abbreviations: UC, unilateral cryptorchidism group; SO, sham-operated group; CRY, cryptorchidism testes; SC, self-control testeis; SO-L, the left testes of sham-operated group.

There was no mortality of mice during the study period, and no surgical complications occurred even though mice were not treated with antibiotics. The left testes of mice in the UC group were found in the abdominal cavity, and the bilateral testes of the SO group and right testes of the UC group were found in the scrotum. The testes and epididymides from UC and SO animals were obtained after the corresponding number of postoperative days. The testes and epididymides of mice in the UC and SO groups were comparable in size, as shown by the image contrast (Fig 1A). The size of the left testes of the UC group decreased significantly from 7 dpo, but the two sides of epididymides showed no obvious changes. Table 1 shows the mean body weight and testicular and epididymis weights of the experimental mice. There were no evident differences in body weights and SC testicular weights among the groups, and the CRY testes of the UC group showed a marked reduction in weight compared with the SC testes at 7, 14, 21 and 28 dpo. However, the left epididymis weight of the UC group (UCE-L) was significantly different to the right epididymis (UCE-R) weight (p < 0.05), but only at 7 dpo (Table 1). Consistent with this, the testes index (ratio of testes weight/body weight) of the CRY testes was reduced compared with the SC and left testes of the SO group (SO-L) at 7, 14, 21 and 28 dpo (Fig 1B). In addition, Kocak I et al.[24] proved that the difference between the left (cryptorchid) and the right epididymis weights was statistically significant at 60 and 90 days after surgery, however, when did its happened was not clear. In our study, we indicated the epididymides index of UCE-L were mild declined was from 21 dpo (Fig 1C), and the cryptorchid epididymis(UCE-L) had a significantly(p<0.05) lower weight than the contralateral ones at 28dpo (Table 1).

**Table 1:** The mean body, testicular and epididymis weights after operation(g).

	UC group					SO group				
	Body weight	Testicular weight		Epididymis weight		Body weight	Testicular weight		Epididymis weight	
		Left (CRY)	Right (SC)	Left (UCE-L)	Right (UCE-R)		Left (SO-L)	Right (SO-R)	Left (SOE-L)	Right (SOE-R)
3d	30.15±3.40	0.1074±0.0132	0.1104±0.0137	0.0274±0.0036	0.0271±0.0033	30.28±1.90	0.1021±0.0141	0.1009±0.0209	0.0272±0.0030	0.0269±0.0030
7d	30.12±2.32	0.0615±0.0192*	0.1022±0.0061	0.0275±0.0022	0.0275±0.0026	29.35±5.42	0.1002±0.0152	0.1029±0.0152	0.0279±0.0039	0.0280±0.0047
14d	35.39±3.72	0.0499±0.0089**	0.1239±0.0850	0.0322±0.0039	0.0335±0.0039	37.88±3.72	0.1367±0.0095	0.1399±0.1072	0.0331±0.0041	0.0336±0.0044
21d	37.53±2.34	0.0702±0.0331*	0.1236±0.0291	0.0330±0.0025	0.0343±0.0029	39.03±2.30	0.1280±0.0100	0.1308±0.0078	0.0347±0.0040	0.0343±0.0041
28d	37.40±3.37	0.0614±0.0287**	0.1291±0.0232	0.0313±0.0022*	0.0371±0.0029	39.94±2.03	0.1397±0.0076	0.1450±0.0093	0.0375±0.0012	0.0377±0.0018

Data show the mean ± s.d., Analysis of paired-samples t test.

**Note:** Testicular and epididymis weights (g). *p < 0.05 and **p < 0.01, compared with the right, respectively. A P-value < 0.05 was considered significant.

**Abbreviations:** UC, unilateral cryptorchidism group; SO, sham-operated group; CRY, cryptorchidism testes; SC, self-control testes; UCE-L, unilateral cryptorchidism epididymis- left; UCE-R, unilateral cryptorchidism epididymis- right; SO-L, the left testes of sham-operated group; SO-R, the right testes of sham-operated group; SOE-L, sham-operated epididymis- left; SOE-R, sham-operated epididymis- right.

**Fig 2:**
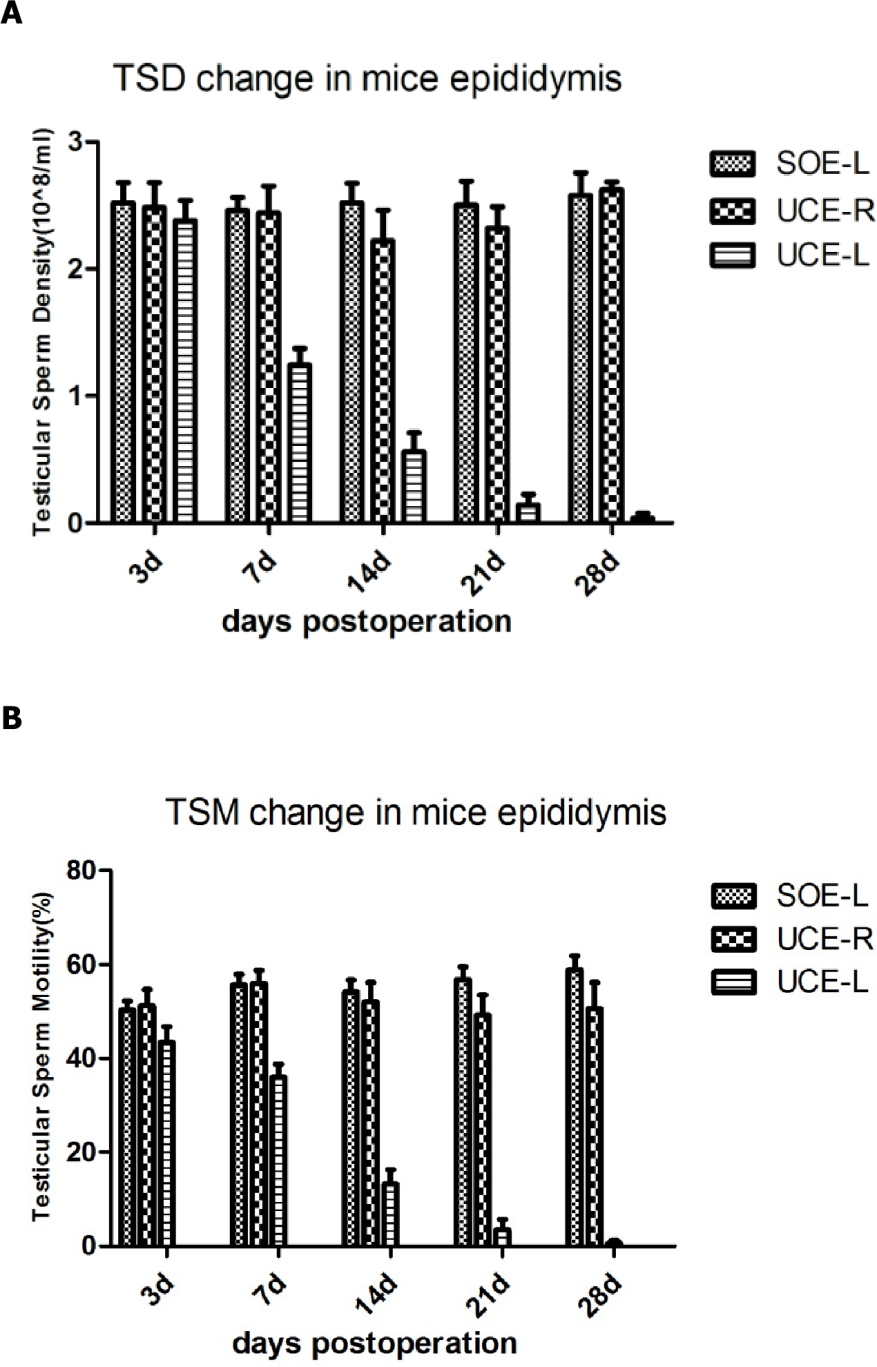
(A) The sperm density change in left side of unilateral cryptorchidism epididymis(UCE-L) compared with UCE-R and SOE-L at different time points. (B) The sperm motility change in left side of unilateral cryptorchidism epididymis(UCE-L) compared with UCE-R and SOE-L at different time points. Note: Results are presented as mean ± s.d. Abbreviations: UCE-L, unilateral cryptorchidism epididymis- left; UCE-R, unilateral cryptorchidism epididymis- right; SOE-L, sham-operated epididymis- left.

The sperm quality outcomes were manually evaluated using the CASA system. We observed changes in the semen quality from the epididymis of experimentally-induced mice, including changes in semen density and motility. The semen analysis was performed by one well-trained technician. The sperm density of the UCE-L showed a marked reduction compared with UCE-R and SOE-L at 7, 14, 21 and 28 dpo (Fig 2A). The sperm motility showed a similar trend (Fig 2B), but there was a slight decrease in the semen motility of UCE-R and SOE-L mice at 3 dpo compared with the other time points, possibly caused by surgical trauma (Fig 2B). There was no difference in the semen quality of SOE-L and SOE-R, indicating no differences between the two sides in the SO group (Tables 2 and 3).

**Table 2:** The comparison of sperm density in bilateral epididymis of experimental mice (10^8/ml).

	UCE		95%CI	t	P	SOE		95%CI	t	P
	L	R				L	R			
3d	2.38±0.36	2.48±0.44	(-0.53,0.33)	-0.645	0.554	2.52±0.36	2.5±0.34	(-0.66,0.70)	0.082	0.939
7d	1.24±0.30	2.44±0.47	(-1.99,-0.41)	-4.216	0.014	2.46±0.23	2.54±0.37	(-0.63,0.47)	-0.403	0.708
14d	0.56±0.34	2.22±0.55	(-2.45,-0.87)	-5.847	0.004	2.52±0.34	2.54±0.42	(-0.77,0.73)	-0.074	0.945
21d	0.14±0.20	2.32±0.38	(-2.64,-1.72)	-13.17	0.000	2.50±0.43	2.48±0.34	(-0.47,0.51)	0.113	0.916
28d	0.04±0.09	2.62±0.15	(-2.76,-2.40)	-38.90	0.000	2.58±0.40	2.52±0.58	(-1.05,1.17)	0.151	0.888

**Table 3:** The comparison of sperm motility in bilateral epididymis of experimental mice (%).

	UCE		95%CI	t	P	SOE		95%CI	t	P
	L	R				L	R			
3d	43.39±7.60	51.26±7.65	(-17.68,1.95)	-2.225	0.090	50.30±4.35	51.42±3.70	51.42±3.70	-0.414	0.700
7d	35.99±6.21	55.99±6.17	(-32.72,-7.29)	-4.37	0.012	55.64±5.07	53.95±8.84	(-12.80,16.19)	0.325	0.762
14d	13.23±6.83	52.01±9.31	(-56.30,-21.24)	-6.14	0.004	54.18±5.66	52.88±6.96	(-3.75,6.34)	0.712	0.516
21d	3.50±4.95	49.22±9.53	(-55.63,-35.80)	-12.8	0.000	56.76±6.20	53.59±6.78	(-10.67,17.01)	0.636	0.559
28d	0.63±1.41	50.67±12.37	(-66.63,-33.44)	-8.371	0.001	58.92±6.47	52.42±6.30	(-8.14,21.14)	1.234	0.285

Data show the mean ± s.d., analysis of paired-samples t test.

**Note:** A P-value < 0.05 was considered significant.

**Abbreviations:** UCE-L, unilateral cryptorchidism epididymis- left; UCE-R, unilateral cryptorchidism epididymis- right; SOE-L, sham-operated epididymis- left; SOE-R, sham-operated epididymis- right; CI = confidence interval.

**Fig 3:**
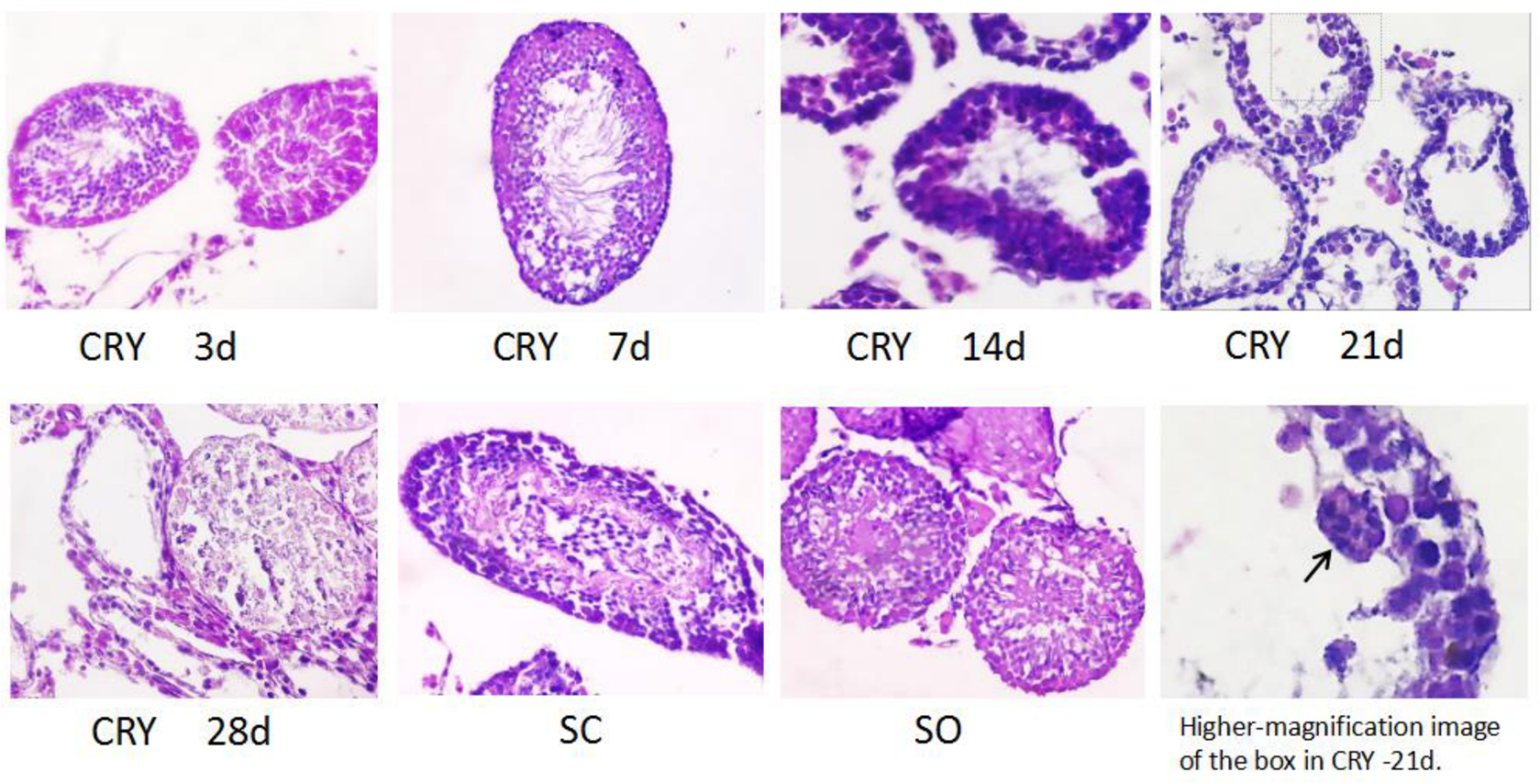
HE analysis of the testes of mice with cryptorchidism. CRY testes shows no differences at 3 dpo compared to the teses of SC and SO. At 7 dpo, the testes with CRY showed internal morphological mild damage and a spot of mature sperm. At 14 dpo, cavitation was observed in the testes of CRY, and with a damaged morphology and lack of mature sperm. At 21 and 28 dpo, both the sperm morphology and internal structure of CRY testes were destroyed, and accompanied with giant multinucleated cells(MNC) formation at 21dpo. Black arrowheads indicate MNCs. Magnification 400× for all images, except where noted. Abbreviations: CRY, cryptorchidism testes; SC, self-control testes; SO, the testes of sham-operated group.

Hematoxylin and eosin staining was carried out to observe the histological changes in the testicles. Morphological comparison of the mature sperm from HE-stained CRY and SC testes at 3 dpo showed no differences compared to those of SO mice (Fig 3). At 7 dpo, the CRY testes showed mild internal morphological damage and a small amount of mature sperm (Fig 3). At 14 dpo, cavitation was observed in the CRY testes, in addition to damaged morphology and a lack of mature sperm (Fig 3). At 21 and 28 dpo, both the sperm morphology and internal structure of the CRY testes were destroyed, accompanied by the presence of giant multinucleated cells (MNC) at 21 dpo (Fig 3). The CRY testes displayed progressive germ cell loss and seminiferous tubule atrophy over the postoperative period. Spermatogenesis did not change significantly in the SC testes compared to the SO testes (Fig 3).

**Fig 4:**
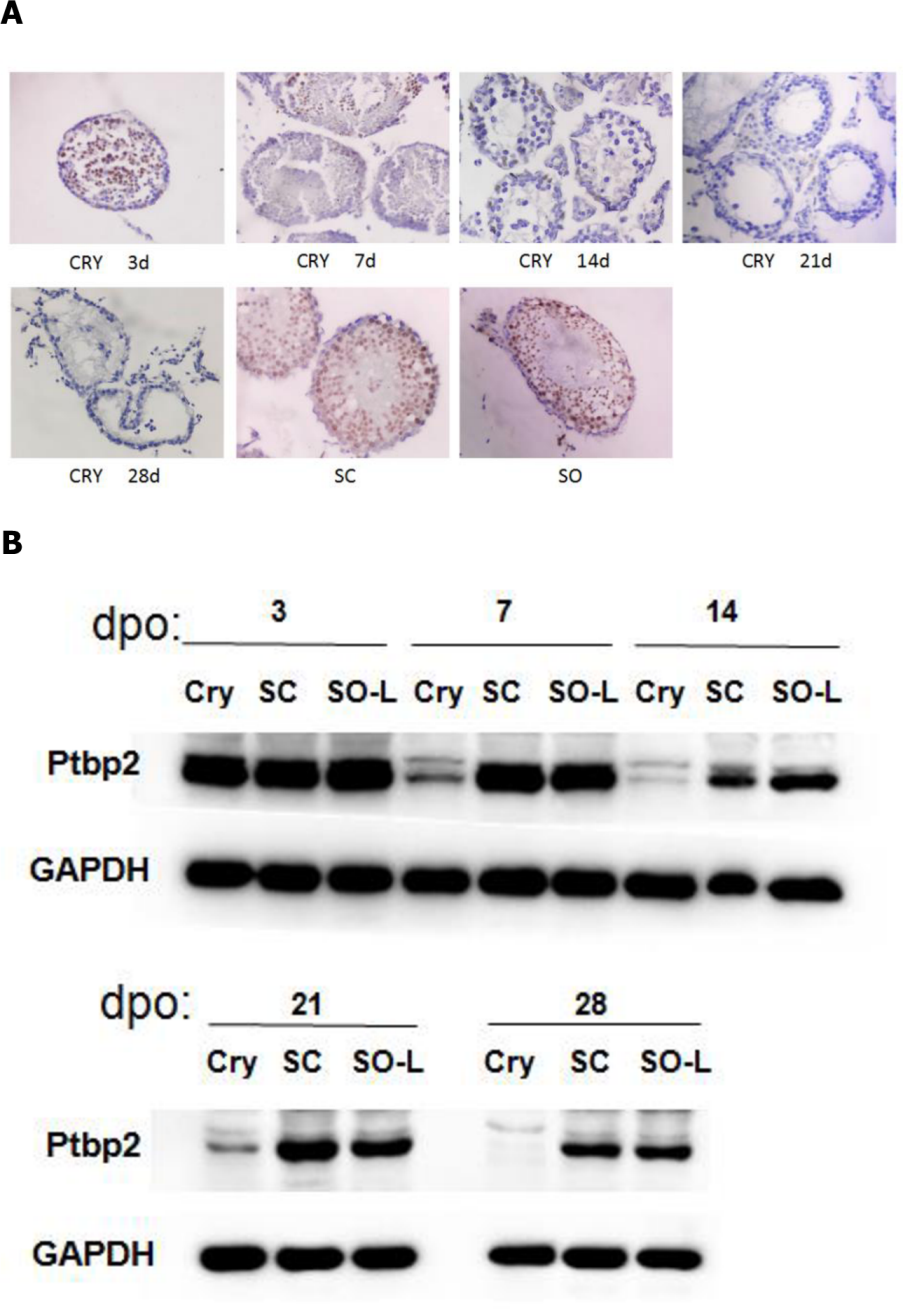
(A) Immunohistochemistry staining testes tissues of mice with cryptorchidism. Magnification 400× for all images. (B) Ptbp2 levels during postoperation testes development. Western blot analysis of mice testes extract prepared from testes collected on the indicated days postoperation (dpo). Abbreviations: CRY, cryptorchidism testes; SC, self-control testes; SO-L, the left testes of sham-operated group; dpo, days postoperation.

Previous studies have demonstrated the presence of Ptbp2 in mice testicular tissues at 5 and 7 days postpartum, and Ptbp2 levels were found to have increased dramatically at 19 days postpartum [13]. Hence, Ptbp2 was highly expressed in 6-week-old male mice. In our experiment, immunohistochemical staining was performed to clarify the location and expression level of Ptbp2 in mice testicular tissues. We found that Ptbp2 was abundantly expressed in all levels of germ cells in the SC and SO testicular tissues (Fig 4A). We then confirmed the high and steady expression of Ptbp2 in SC and SO-L testicular tissues by western blot analysis (Fig 4B), except for a slight decrease in expression observed at 14 dpo compared with that found at 3 and 7 dpo, possibly because fewer loading quantity of protein lysates were analyzed at this time-point. In CRY testes, Ptbp2 was significantly decreased by 7 dpo, which was consistent with reduction of the testis index, and was barely detected thereafter (Fig 4A and 4B).

**Fig 5:**
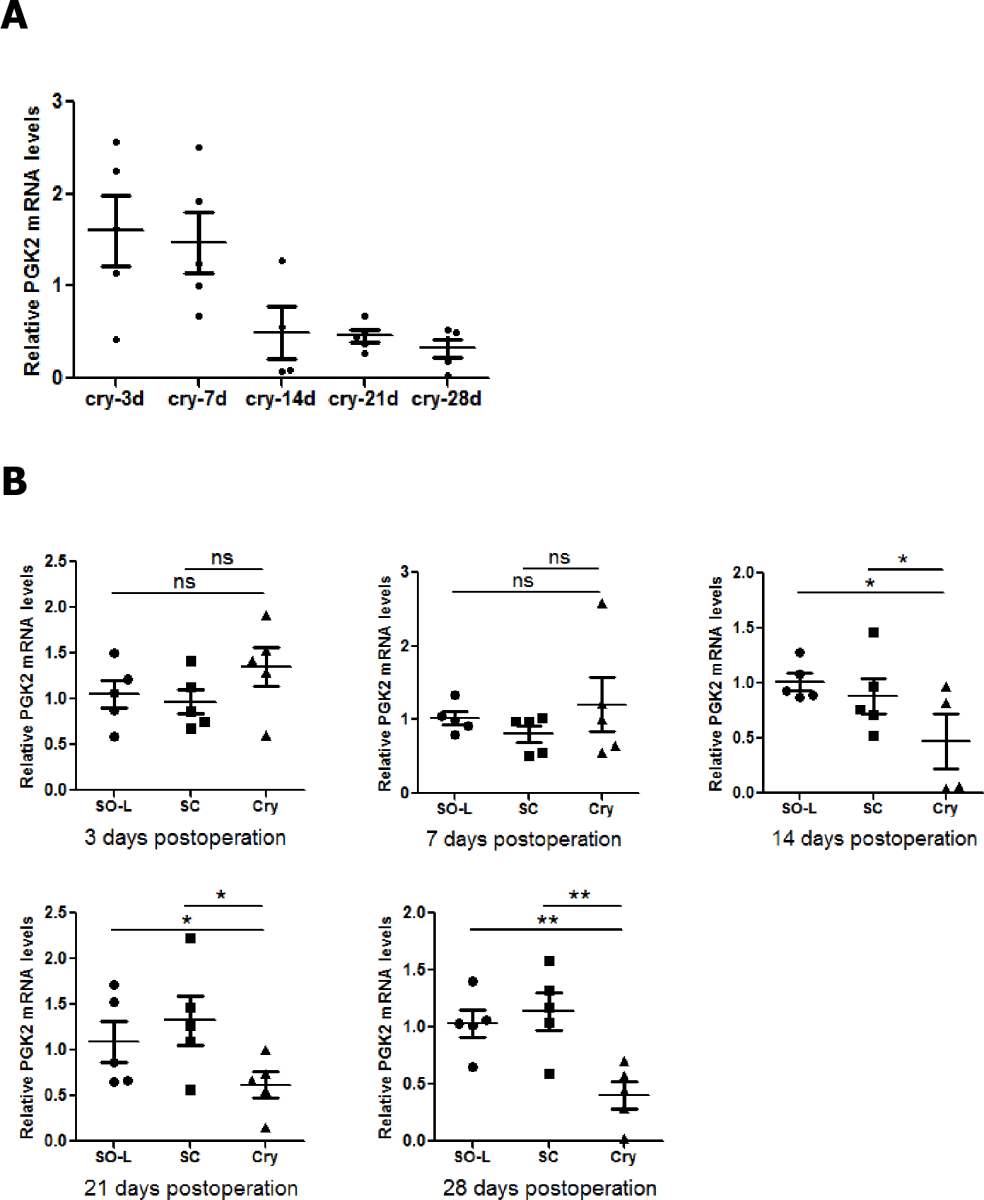
(A) Quantitative real-time PCR analysis of PGK2 mRNA expression levels in different time period(When they reached days 3,7,14,21 and 28 after operation) mice testes tissues of cryptorchidism(CRY) group. (B) The PGK2 mRNA expression levels change in CRY testes compared with SC and SO-L at different time points. Note: *p < 0.05, **p < 0.01. A P-value < 0.05 was considered significant. Abbreviations: mRNA, messenger RNA; PGK2, phosphoglycerate kinase 2; cry, cryptorchidism group testes; SC, self-control testes; SO-L, the left testes of sham-operated group; ns, no significant(p >0.05).

Based on previous results from *in vitro* cell culture assays with synthetic RNA, Ptbp2 has been proposed to play a role in the stabilization of Pgk2 transcripts [16,25]. To investigate whether the decrease in Ptbp2 affected Pgk2 mRNA expression levels *in vivo*, quantitative real-time PCR was employed to detect the Pgk2 transcript levels in UC and SO testes. The Pgk2 mRNA expression levels were analyzed. Relative to β-actin mRNA, there were no marked changes in the Pgk2 mRNA levels in the CRY testicular tissues at 3 and 7 dpo. However, Pgk2 mRNA expression was significantly decreased at 14, 21 and 28 dpo (Fig 5A and 5B).

## Discussion

Many models for the induction of cryptorchidism in experimental animals have been described in the literature. In mice, cryptorchidism is usually induced by endocrine [26] or mechanical [2] methods. Congenital cryptorchid mutant rats have also been used as a natural model [27]. It is known that appropriate temperature is needed for spermatogenesis, especially proliferation and differentiation of germ cells is temperature dependent, requiring a temperature of at least 1–2°C lower than body temperature. Germ cell injury induced in the cryptorchidism testes is caused mainly by exposure of the testes to a relatively high abdominal temperature. Therefore, we generated a cryptorchidism model in mice by a surgical procedure, which is predominantly used to investigate the molecular mechanisms of germ cell injury resulting from heat stress due to the abdominal placement.

In unilateral cryptorchidism mice, we report compelling evidence that the sperm cells were severely damaged, in addition to non-obstructive azoospermia. Our results for changes in the mean testes weight and testes index indicated continual atrophy of the CRY testes in the high intra-abdominal position. These results are in accordance with the results of Zakaria et al. [28]. Furthermore, HE staining was carried out to evaluate the testicular histological changes. The CRY testes displayed progressive germ cell injury and seminiferous tubule atrophy during the postoperative period [29]. Furthermore, the presence of giant MNC indicates apoptotic features of the germ cells. Moreover, we measured the sperm density and motility using the WLJY-9000 CASA, one of the branded CASA systems commonly used in andrology laboratories in China [30]. There was no significant difference in the semen quality of the SO group, consistent with the findings for the control groups in studies by Kim et al. [31] and Bae et al. [32]. In addition, sperm was rarely detected in UCE-L at 21 and 28 dpo, indicating non-obstructive azoospermia, which is the one of the most serious complications associated with cryptorchidism.

To examine the specific mechanism underlying the role of Ptbp2 in the process of heat stress-induced germ cell injury, western blotting and IHC was performed to visualize and measure Ptbp2 expression levels. In the present study, Ptbp2 was found to be reduced in CRY testes at 7 dpo, and was barely detected thereafter. To investigate whether the decrease in Ptbp2 affected Pgk2 mRNA abundance, quantitative real-time PCR was performed to confirm the role of Ptbp2 in the stabilization of germ cell Pgk2 mRNA, as previously described [33]. The mRNA that encodes the testes-specific protein phospho-glycerate kinase (Pgk2) is a long-lived mRNA that is transcribed in meiotic and postmeiotic male germ cells. Pgk2 mRNA is present in germ cells for up to 2 weeks before its protein product is detected. Coimmunoprecipitation experiments confirmed that Ptbp2 binds to the 3'-UTR of the Pgk2 mRNA in the testes [16]. In this study, the Pgk2 mRNA expression levels decreased significantly by 14 dpo, which was later than the decline in Ptbp2. Based on the results of several other studies, this indicates that Ptbp2 binds to Pgk2 mRNA sequences in male murine germ cells [16,19,20]. We conclude that Ptbp2 is an essential factor for the stabilization of Pgk2 mRNA in testicular tissue.

In summary, a significant decline in Ptbp2 and germ cell injury was observed in the testes of mice with UC group. We hypothesize that Ptbp2 is susceptible to heat stress and its disruption has resulted in stability decline of germ cell transcripts Pgk2 mRNA, which consequently lead to germ cell injury in cryptorchidism testes. If left no intervention measure to shield this crucial signaling pathways of spermatogenesis, non-obstructive azoospermia and testicular atrophy are serious complications of intraperitoneal cryptorchidism.

The preliminary research described here has partially revealed the function of Ptbp2, and confirmed the relationship between Ptbp2 and germ cell injury. Importantly, this study is the first to confirm the relationship between Ptbp2 and germ cell injury in cryptorchidism. Further experiments on the causal link between Ptbp2 and germ cell injury is required due to our insufficient knowledge regarding the etiology of cryptorchidism-induced infertility. We expect that individualized treatment could be implemented to delay damage to the sperm cells of cryptorchidism patients prior to orchiopexy. Furthermore, it is important to note that we did not investigate the function of Ptbp2 in invitro germ cell and cryptorchidism patients, and we plan to do this in future research. Cryptorchidism-induced infertility is considered to be a disease with a complex etiology in humans [34–36], and further investigation in this field is needed.

## Supporting information

S1 Table The relative expression of Pgk2 mRNA. (XLSX)

S2 Table The sperm density and motility data. (XLSX)

S3 Table The body、testicular and epididymis weights. (XLSX)
